# Understanding the Effects of Both CD14-Mediated Innate Immunity and Device/Tissue Mechanical Mismatch in the Neuroinflammatory Response to Intracortical Microelectrodes

**DOI:** 10.3389/fnins.2018.00772

**Published:** 2018-10-31

**Authors:** Hillary W. Bedell, Sydney Song, Xujia Li, Emily Molinich, Shushen Lin, Allison Stiller, Vindhya Danda, Melanie Ecker, Andrew J. Shoffstall, Walter E. Voit, Joseph J. Pancrazio, Jeffrey R. Capadona

**Affiliations:** ^1^Department of Biomedical Engineering, School of Engineering, Case Western Reserve University, Cleveland, OH, United States; ^2^Advanced Platform Technology Center, L. Stokes Cleveland VA Medical Center, Rehab. R&D, Cleveland, OH, United States; ^3^Department of Bioengineering, The University of Texas at Dallas, Richardson, TX, United States; ^4^Center for Engineering Innovation, The University of Texas at Dallas, Richardson, TX, United States; ^5^Department of Materials Science and Engineering, The University of Texas at Dallas, Richardson, TX, United States; ^6^Department of Mechanical Engineering, The University of Texas at Dallas, Richardson, TX, United States

**Keywords:** intracortical microelectrodes, neuroinflammation, innate immunity, softening electrode, shape memory polymer

## Abstract

Intracortical microelectrodes record neuronal activity of individual neurons within the brain, which can be used to bridge communication between the biological system and computer hardware for both research and rehabilitation purposes. However, long-term consistent neural recordings are difficult to achieve, in large part due to the neuroinflammatory tissue response to the microelectrodes. Prior studies have identified many factors that may contribute to the neuroinflammatory response to intracortical microelectrodes. Unfortunately, each proposed mechanism for the prolonged neuroinflammatory response has been investigated independently, while it is clear that mechanisms can overlap and be difficult to isolate. Therefore, we aimed to determine whether the dual targeting of the innate immune response by inhibiting innate immunity pathways associated with cluster of differentiation 14 (CD14), and the mechanical mismatch could improve the neuroinflammatory response to intracortical microelectrodes. A thiol-ene probe that softens on contact with the physiological environment was used to reduce mechanical mismatch. The thiol-ene probe was both softer and larger in size than the uncoated silicon control probe. *Cd14^-/-^* mice were used to completely inhibit contribution of CD14 to the neuroinflammatory response. Contrary to the initial hypothesis, dual targeting worsened the neuroinflammatory response to intracortical probes. Therefore, probe material and CD14 deficiency were independently assessed for their effect on inflammation and neuronal density by implanting each microelectrode type in both wild-type control and *Cd14^-/-^* mice. Histology results show that 2 weeks after implantation, targeting CD14 results in higher neuronal density and decreased glial scar around the probe, whereas the thiol-ene probe results in more microglia/macrophage activation and greater blood–brain barrier (BBB) disruption around the probe. Chronic histology demonstrate no differences in the inflammatory response at 16 weeks. Over acute time points, results also suggest immunomodulatory approaches such as targeting CD14 can be utilized to decrease inflammation to intracortical microelectrodes. The results obtained in the current study highlight the importance of not only probe material, but probe size, in regard to neuroinflammation.

## Introduction

Intracortical microelectrodes allow researchers to record single-unit and multi-unit activity from individual or groups of neurons by detecting changes to the extracellular potential as a result of neurons generating action potentials (Renshaw et al., 1940; Wessberg et al., 2000). Recorded neural signals afford neuroscientists insight into the activity of specific populations of neurons. Thus, intracortical microelectrodes provide a valuable research tool to the field of cognitive and sensorimotor neuroscience. Intracortical microelectrodes are also utilized in brain–computer interfacing (BCI) applications to record neural activity as an input signal to decode and extract motor intent (Schwartz, 2004; Hochberg et al., 2012). Recorded neural signal informs the generation of a desired action for an external device, prosthetic, or muscles (*via* muscle stimulators) for a patient suffering from paralysis or limb loss. Thus, intracortical microelectrodes are a promising technology for both basic research and the development of clinical neuroprosthetic devices.

For both clinical and research applications, intracortical microelectrodes must be able to record from single cortical neurons for long periods (months to years). Unfortunately, there are limitations to intracortical microelectrodes that impede device reliability. Many studies document the failure of intracortical microelectrodes exemplified by both decrease of signal to noise ratio and loss of number of channels detecting single units (Polikov et al., 2005; Liu et al., 2006; Rennaker et al., 2007; Barrese et al., 2013). There are multiple factors that contribute to the failure of intracortical microelectrodes, including but not limited to a biological response to chronically implanted intracortical microelectrodes (Rennaker et al., 2007; Saxena et al., 2013; Kozai et al., 2014b; Hermann et al., 2017).

Inflammation ensues after the device damages tissue during implantation when blood vessels are unavoidably severed leading to blood infiltration and serum protein adsorption onto the device. Implantation results in the release of endogenous damage signals such as high mobility growth box 1 (HMGB1) and inflammatory lipids from damaged cells (Potter et al., 2014). Plasma proteins and damage-associated molecular patterns (DAMPs) are recognized by cellular receptors such as the toll-like receptor (TLR)/cluster of differentiation 14 (CD14) complex. As a result, microglial and infiltrating macrophage cells become inflammatory or “activated” and subsequently release of pro-inflammatory molecules (Kim S. et al., 2013; Zanoni et al., 2017). Glial encapsulation, neurodegeneration, and neuronal death follow this inflammatory cascade. Since the long-term success of the devices depends on the presence of healthy, active neurons immediately adjacent to the recording sites of the probe, the inflammatory process leads to a reduction of detectable signals necessary for BCI and other neuroscience research applications (Schwartz, 2004; Bjornsson et al., 2006; Jorfi et al., 2015).

In addition to the primary injurious events caused by the initial implantation, a persistent inflammatory response is present at the probe–tissue interface under chronic conditions. The pro-inflammatory microenvironment resulting from probe implantation leads to further breakdown of the blood–brain barrier (BBB) and increased vascular permeability perpetuating the inflammatory cascade (Abdul-Muneer et al., 2015).

Furthermore, the mechanical mismatch between a traditional probe (with a metal or silicon substrate) and the brain can exacerbate inflammation (Harris et al., 2011; Moshayedi et al., 2014; Nguyen et al., 2014; Du et al., 2017; Lee et al., 2017a). The mechanical discrepancy in modulus between the non-compliant probe and the pliant brain results in tissue strain and compression at the tissue–device interface. We have shown that reducing the modulus of the probe from 100 to 1000 s of GPa, to 1–10 s of MPa to more closely match that of gray matter in brain tissue (*E* = ∼3–6 kPa (Green et al., 2008)) reduces the micromotion-induced strain (Sridharan et al., 2015). As a result, after implantation of such relatively compliant materials, the inflammatory response to intracortical microelectrodes is significantly reduced, but not completely eliminated, providing neuroprotection and a more stable BBB (Harris et al., 2011; Nguyen et al., 2014).

Increased BBB permeability can facilitate the infiltration of myeloid cells into the injured brain tissue. These peripheral immune cells become activated and perpetuate the inflammatory response leading to neuronal death by the probe–tissue interface (Ravikumar et al., 2014). To combat the inflammatory response from microglia and infiltrating myeloid cells triggered by recognition of DAMPs and serum proteins, our lab has explored targeting the TLR/CD14 pathway involved in the recognition of DAMPs as a method to improve the chronic recording performance and reduce inflammation around the brain–electrode interface (Hermann et al., 2017). More recently, we have also shown that targeting the TLR/CD14 pathway in only infiltrating blood-derived cells leads to an improvement in chronic recording quality (Bedell et al., 2018).

Our initial softening probes used in the Capadona Lab yielded desirable mitigation of the neuroinflammatory response to intracortical microelectrodes (Capadona et al., 2008; Nguyen et al., 2014). However, these initial materials swelled up to 70% in aqueous conditions, making fabrication into functional electrodes problematic. In collaboration with the Voit and Pancrazio labs, we have begun exploring similar softening materials, thiol-ene and thiol-ene/acrylate shape memory polymers (softening from ∼1.7 GPa down to ∼35 MPa (Ecker et al., 2017)) that possess fabrication benefits over the initial nanocomposite softening probes. These polymers soften under physiological conditions due to plasticization effects, but swell only up to 3%. The strong interaction between thiols and noble metals commonly used for electrodes yields improved adhesion between substrate and thin film metals (Nuzzo et al., 1987). Moreover, thiol-ene and thiol-ene/acrylates are more compatible with high yield, high-resolution photolithographic processes enabling manufacturability. Most importantly, a functional device, comprised of a thiol-ene/acrylate substrate, has been synthesized which was able to record single units for more than 2 months (Simon et al., 2017). Therefore, in the current study, thiol-ene was used as a probe substrate material that more closely matches the modulus of the brain to reduce BBB breakdown while also targeting an innate immune pathway involved in the recognition of serum proteins. Our work combines these approaches using a softening material and targeting CD14 through *Cd14^-/-^* mice, to reduce neuroinflammation in response to single-shank intracortical microelectrode probes.

## Results

The current study aimed to determine whether the dual targeting of the innate immune response and the mechanical mismatch between tissue and a single-shank probe, which generates tissue damage, results in combinatorial or synergistic effects to improve neuronal density and reduce inflammation at the probe–tissue interface. We utilized a thiol-ene probe, which is stiff when inserted, but softens at physiological temperatures as the probe that more closely matches the brain tissue modulus. A *Cd14^-/-^* knock-out model was used to target the innate immune response while immunohistochemistry was used to evaluate the neuroinflammatory response. A neuronal nuclear protein, NeuN, was used as an immunohistochemical marker for cortical neurons around the probe (Mullen et al., 1992). The glial scar is an indicator of neuroinflammation, so glial fibrillary acidic protein (GFAP), a type of intermediate filament protein upregulated by reactive astrocytes, was examined *via* immunohistochemistry (Landis, 1994). To explore microglia and macrophage activation, an antibody to CD68, was used to detect activated microglia/macrophages around the probe interface. CD68 is an lysosomal-associated membrane protein which may play a role in antigen processing and presenting (Song et al., 2011). Furthermore, BBB dysfunction characterizes inflammation resulting from a neural implant such as an intracortical probe. BBB disruption was evaluated by quantifying the presence of IgG, a prolific plasma protein not found in the brain parenchyma under normal conditions, using an anti-IgG antibody (Potter et al., 2012a).

### Comparing Dual Innate Immune Response and Mechanical Mismatch to Control

**FIGURE 1:**
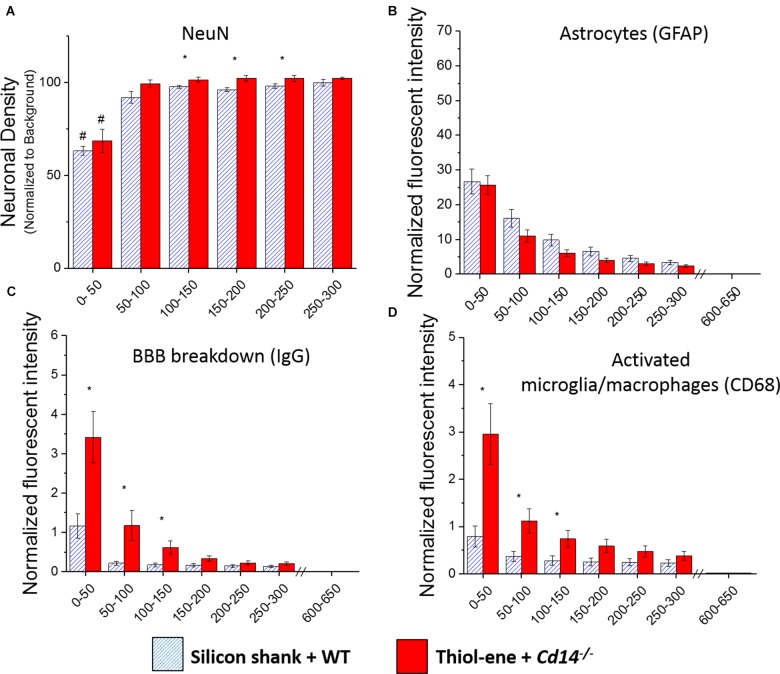
Immunohistochemical evaluation comparing the dual targeting of the innate immune response and mechanical mismatch to control at 2 weeks after implantation. All analyses were evaluated with respect to distance from the explanted microelectrode hole (μm). **(A)** Neuronal density evaluated as NeuN^+^ cells.**(B)** Astrocyte encapsulation evaluated as GFAP expression. **(C)** Blood-brain barrier permeability evaluated as IgG expression. **(D)** Microglial and macrophage activation evaluated as CD68 expression. ^∗^ Denotes significance between silicon shank + WT and thiol-ene + *Cd14^-/-^*; ^#^ denotes significant difference from background neuronal density.

We first aimed to determine whether knocking out CD14 while using a softening probe would lead to reduced neuroinflammation and improved neuronal density around the probe at 2 weeks post implantation. For both experimental and control conditions, neuronal density at 0–50 μm from the implant surface was significantly lower than that at the background (300–350 μm from probe surface). Additionally, neuronal density surrounding softening (thiol-ene) probes in *Cd14^-/-^* animals was significantly higher than control animals at each 50 μm interval from 100 to 250 μm from the probe surface (Figure 1A). There was no difference in glial scar between these two conditions at any distance interval from the probe (Figure 1B). However, the combinatorial targeting approach increases BBB disruption and activated macrophages and microglia at each 50 μm interval from 0 to 150 μm from the probe (Figures 1C,D). Altogether, these data suggest that dual targeting the innate immune response while using the thiol-ene softening probe actually worsens the neuroinflammatory response. Thus, subsequent experiments were conducted to delineate the role of each strategy – targeting CD14 versus using a thiol-ene probe.

### Delineating Effect of Each Variable – Probe Stiffness and CD14 Expression

To elucidate if either factor (probe stiffness or CD14 expression) drove the increased neuroinflammatory response of the combinatorial targeting, additional animals were set up as controls for each factor resulting in four different conditions (silicon shank + WT, silicon shank + *Cd14^-/-^*, thiol-ene + WT, thiol-ene + *Cd14^-/-^*). Additional animals were also set up to examine more chronic (16 weeks post-implantation) time points for each of the four experimental conditions. Comparisons were made between levels of each independent variable – responses of softening thiol-ene probes versus stiff silicon probes and comparisons between *Cd14^-/-^* and wild-type animals.

#### Neuronal Density

**FIGURE 2:**
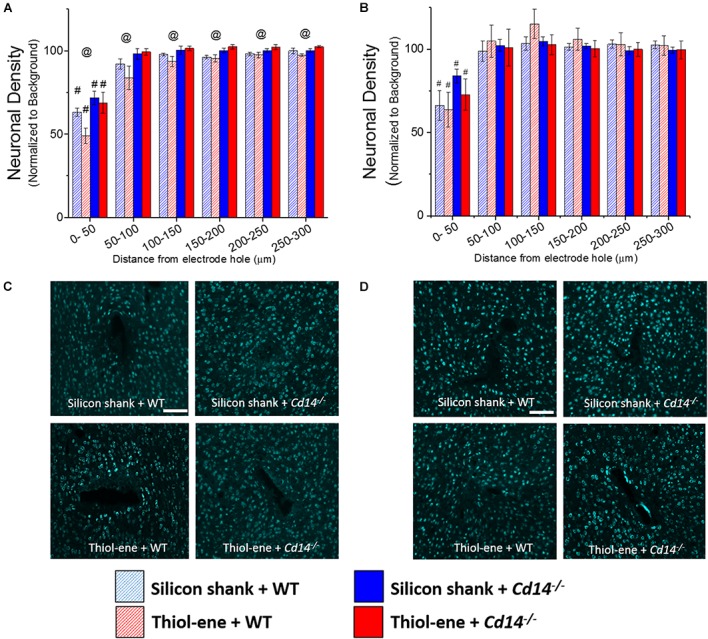
Immunohistochemical evaluation of neuronal density. Neuronal density evaluated as NeuN^+^ cells with respect to distance from the explanted microelectrode hole (μm). **(A)** 2 weeks. **(B)** 16 weeks. **(C)** Representative images of 2 weeks neuronal density. **(D)** Representative images of 16 weeks neuronal density. Scale bar: 100 μm. ^@^ Denotes significance between WT and *Cd14^-/-^*; ^#^ denotes significant difference from background neuronal density.

At both 2 and 16 weeks post implant, neuronal density at 0–50 μm from the electrode surface was significantly lower than that at the background for all conditions regardless of substrate material or genotype (Figures 2A,B). In the absence of CD14, neuronal density at each interval from 0 to 300 μm was significantly higher than that of wild-type at 2 weeks post implant (Figures 2A,C). However, at the chronic time point, 16 weeks, CD14 deficiency seemed to play a lesser role as there were no significant differences between wild-type and *Cd14^-/-^* animals (Figures 2B,D). At both 2 and 16 weeks post implant, the thiol-ene probe did not result in increased neuronal density compared to the control silicon probe (Figure 2). Furthermore, the combinatorial approach of using a softening substrate and targeting CD14 did not significantly improve neuronal density over the other conditions (Figure 2). Thus, targeting CD14 results in higher neuronal density around the probe at 2 weeks, but combining CD14 inhibition with a reduction in probe stiffness was counterproductive.

#### Glial Scarring

**FIGURE 3:**
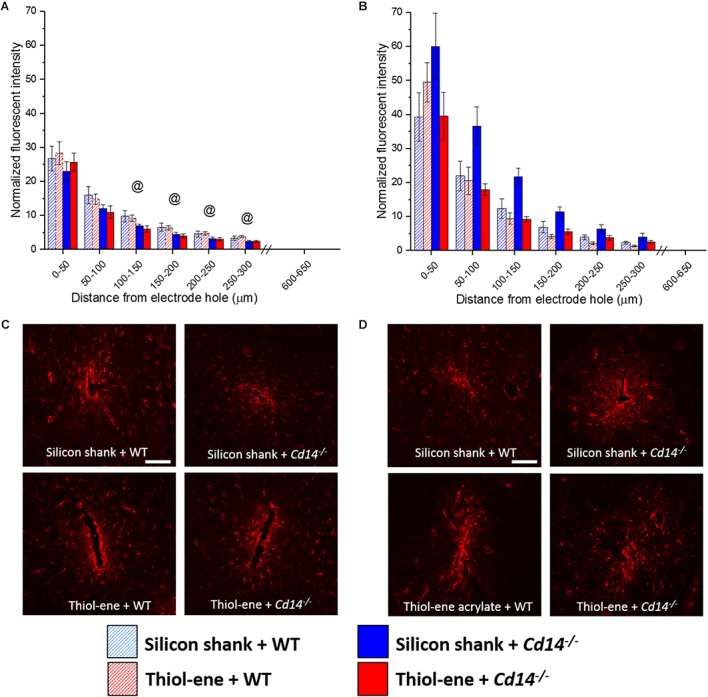
Immunohistochemical evaluation of glial scarring assessed *via* astrocyte encapsulation. Astrocyte encapsulation evaluated as GFAP expression with respect to distance from the explanted microelectrode hole (μm). **(A)** 2 weeks. **(B)** 16 weeks. **(C)** Representative images of 2 weeks glial scar. **(D)** Representative images of 16 weeks glial scar. Scale bar: 100 μm. ^@^ Denotes significance between WT and *Cd14^-/-^*.

In all conditions, the glial scar was the densest closest to the probe and decreased as a function of distance from the probe–tissue interface (Figure 3). The glial scar became denser from 2 to 16 weeks post-implantation for all conditions (Figure 3), indicated by more intense GFAP staining from 0 to 50 μm from the surface of the implants. In animals that do not express CD14, there is less glial scar compared to wild-type animals at each 50 μm interval from 100 to 300 μm from the probe-tissue interface at 2 weeks post-implantation (Figures 3A,C), but not 16 weeks post-implantation (Figures 3B,D). Notably, there were no significant differences in the glial scar between the thiol-ene probe and control silicon probe at either time point (Figure 3). To summarize, targeting CD14 decreases glial scar around probe at 2 weeks.

#### Microglia/Macrophage Expression

**FIGURE 4:**
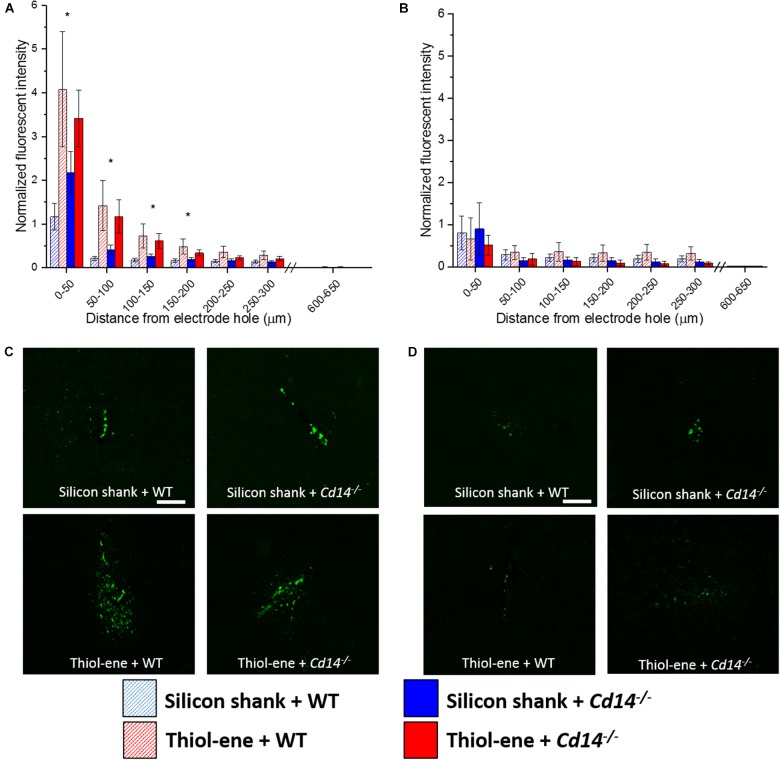
Immunohistochemical evaluation of activated microglia and macrophages. Microglial and macrophage activation evaluated as CD68 expression with respect to distance from the explanted probe hole (μm). **(A)** 2 weeks. **(B)** 16 weeks. **(C)** Representative images of 2 weeks activated microglia and macrophages. **(D)** Representative images of 16 weeks activated microglia and macrophages. Scale bar: 100 μm. ^∗^ Denotes significance between silicon and thiol-ene probes.

Overall, microglial/macrophage activation as assessed *via* CD68 expression was heavily increased at the probe–tissue interface, and declined to background levels (zero expression) as a function of distance from the interface (Figure 4). The thiol-ene probes resulted in more activated microglia/macrophages than the silicon probes at 2 weeks post-implantation at each interval 0–200 μm from the probe–tissue interface (Figures 4A,C). However, there were no significant differences between wild-type and *Cd14^-/-^* groups at 2 weeks post-implantation (Figures 4A,C). By 16 weeks post-implant, activated microglia/macrophages for all groups had decreased compared to 2 weeks post-implantation (Figures 4A,B). Additionally, there were no differences in activation of microglia/macrophages among the conditions at 16 weeks (Figures 4B,D). Altogether, thiol-ene probe results in more microglia/macrophage activation around the probe at 2 weeks, but not 16 weeks post-implantation.

#### BBB Disruption

**FIGURE 5:**
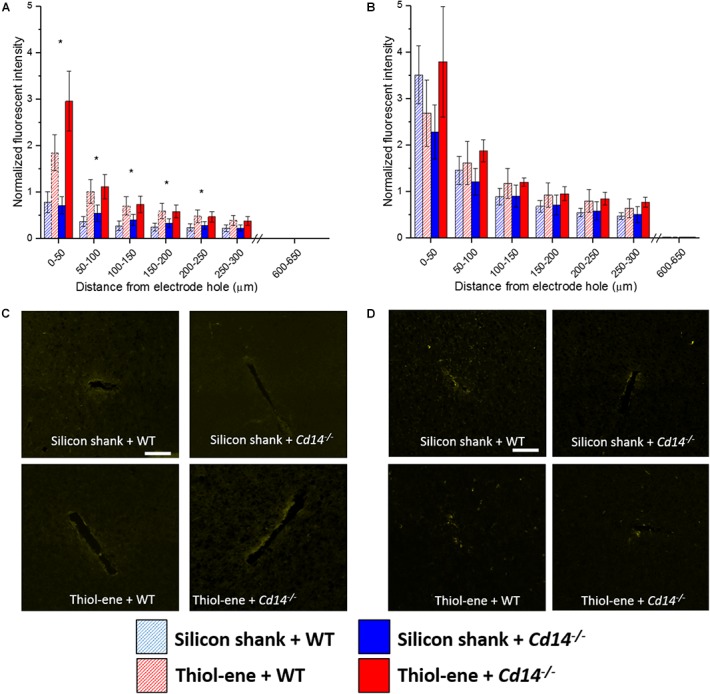
Immunohistochemical evaluation of blood-brain barrier permeability. Blood–brain barrier permeability evaluated as IgG expression with respect to distance from the explanted microelectrode hole (μm). **(A)** 2 weeks. **(B)** 16 weeks. **(C)** Representative images of 2 weeks BBB permeability. **(D)** Representative images of 16 weeks BBB permeability. Scale bar: 100 μm. ^∗^ Denotes significance between silicon and thiol-ene probes.

Similar to the glial scar and activated microglia/macrophages, BBB disruption (IgG expression) was found to be greatest at the probe-tissue interface and decreased in intensity as distance from probe-tissue interface increased (Figure 5). The only significant differences found between the groups were at 2 weeks post-implantation (Figures 5A,C). At 2 weeks post-implantation, the softer thiol-ene probes yielded significantly greater BBB breakdown compared to the stiff silicon probes (at each interval examined between 0 and 250 μm from probe interface, Figures 5A,C). In summary, thiol-ene probe results in greater BBB disruption around the probe at 2 weeks.

## Discussion

The current study explores how a softening thiol-ene probe and/or targeting the TLR/CD14 innate immune pathway affects inflammation and neuronal density at both 2 and 16 weeks post-implantation of intracortical microelectrode probes. Because initial results exploring synergistic effects of two different approaches resulted in increased neuroinflammation, we set out to parse out the response of each variable to gain a better understanding of each strategy alone. Our results demonstrate targeting CD14 results in higher neuronal density and decreased astroglial scarring around the probe at 2 weeks (Figures 2A,C, 3A,C). We also describe the use of the thiol-ene probe with a modulus 3 orders of magnitude lower and a cross-sectional area 4× greater than the control silicon probes. Our observations demonstrate that a probe with a lower modulus but larger implantation footprint resulted in more BBB breakdown and activated microglia/macrophages than the control silicon probes. However, in spite of these markers of inflammation, the softening thiol-ene probes did not result in significantly decreased neuronal density or increased astroglial scarring around the implant at either acute (2 weeks) or chronic (16 weeks) time points.

Physiological sources such as respiration and vascular pulsations result in micromotion of an intracortical probe against brain tissue resulting in strain on brain tissue which can induce tissue damage (Subbaroyan et al., 2005; Gilletti and Muthuswamy, 2006). One of the approaches to reduce the effects of micromotion is to increase probe flexibility. Decreasing the modulus of the probe material is one commonly explored/hypothesized methods to increase flexibility of the device. Probes made of polymers with a modulus or overall stiffness closer to brain tissue such as PDMS, polyimide, and SU-8 have been explored (Rousche et al., 2001; McClain et al., 2011; Altuna et al., 2013). However, implanting such soft probes present challenges with their insertion into the brain. A probe with too soft of a modulus will buckle during insertion and compress the brain tissue during the insertion process (Hess et al., 2013; Jorfi et al., 2015). Current methods used to implant soft electrodes while avoiding buckling include temporarily increasing the stiffness of the probe by use of a coating that dissolves after implantation or a stiff shuttle that accompanies the soft probe and is later removed (Lewitus et al., 2011; Kim B.J. et al., 2013; Kozai et al., 2014a; Vitale et al., 2015). Unfortunately, both approaches increase the footprint of the primary implant which can exacerbate the acute damage to brain tissue during implantation and lead to an increase in inflammation.

Materials which are innately stiff enough to implant, but soften while residing in tissue minimize the modulus difference between probe and brain tissue. Previous literature has suggested that a softening material can compensate for increased damage footprint generated by a device with a larger cross-sectional area compared to a stiff silicon control probe (Nguyen et al., 2014). However, we found this phenomenon to be inconsistent in the current study as the larger thiol-ene probes had a larger cross-sectional area and resulted in more activated microglia/macrophages and increased evidence of BBB breakdown at 2 weeks compared to the smaller, stiffer silicon probes (Figures 4A,C, 5A,C).

The thiol-ene probes used in the current study had a cross-sectional area of about 9000 μm^2^, which is a 4× increase over the silicon probes utilized. The bending stiffness of the probe is determined by both the Young’s modulus (*E*) and the probe dimensions. Although modulus of the probe material affects flexibility of the device, the probe dimensions play more of a role in reducing stiffness; bending stiffness is proportional to *Et*^3^, where *t* is the cross-sectional area of the probe (Salatino et al., 2017).

A larger implant confers more acute tissue damage during implantation leading to greater extravasation of blood cells and proteins (Saxena et al., 2013). However, Nguyen et al. showed that a polyvinyl acetate, another softening material, can overcome increased inflammation induced by a slightly larger penetration profile. The polyvinyl acetate probes used in Nguyen et al. (2014) had a pre-implant cross-sectional area that was 1.5× larger than control polyvinyl acetate dip-coated silicon probes. However, findings by Nguyen et al. (2014) were not recapitulated with thiol-ene, as the thiol-ene resulted in more activated microglia and macrophages and increased BBB permeability compared to the silicon probes (Figures 4, 5). Therefore, there might be a size threshold to where a probe comprised of a softer material cannot overcome the increased inflammation resulting from an increased penetration profile.

The results from the current study suggest that the degree of initial trauma influences acute but not chronic inflammatory response, as there were significant results at 2 weeks post implantation, but there were no differences in the inflammatory response or neuronal density at 16 weeks between implants of different sizes (Figures 2B, 3B, 4B, 5B). Current findings are consistent with Szarowski et al. (2003) which suggests initial glial response is correlated with the cross-sectional area of the electrode; however, sustained response of the implant was independent of probe size.

The different swelling properties of thiol-ene and polyvinyl acetate could also elicit differences in inflammation. The polyvinyl acetate swells ∼70% by volume under physiological conditions (Nguyen et al., 2014), while the thiol-ene substrate yields very minimal (<3%) swelling in physiologic environment (Ware et al., 2014). From a fabrication standpoint, minimal swelling is desirable as fluid uptake from the substrate results in cracking of thin film conductors used for the electrodes. However, as posited by Skousen et al. (2015), the thick hydrogel formed by the swollen polyvinyl acetate could have functioned as a sink for pro-inflammatory cytokines and chemokines at the probe–tissue interface, both of which facilitate inflammation. Reduced pro-inflammatory molecules at the device interface can lead to decreased inflammation and neuronal death. Future studies will need to explore the aforementioned theory.

In the current study, the thiol-ene probe resulted in an increased inflammatory response, likely because of its greater surface area and vascular damage during implantation, consistent with previously published research (Karumbaiah et al., 2013; Lee et al., 2017b; Spencer et al., 2017). The thiol-ene materials utilized in the present study have previously undergone testing to control for material surface chemistry. Shoffstall et al. (2018a) demonstrated that intracortical silicon probes dip-coated with the SMP material (SI, Section “Materials and Methods”) generated similar histological responses (with respect to neuronal survival, activated microglia/macrophages, and BBB permeability) as compared to size-matched bare-silicon probes. While there was a statistically significant reduction in reactive astrocyte staining (GFAP) with the dip-coated probes, taken by itself it is unclear if the effects are substantially different from the bare silicon probes. Similar results suggestive of the effects of cross-sectional dimensions are found using a chemistry-controlled experiment (Supplementary Figure S1). Thiol-ene probes with larger cross-sectional width were compared to silicon probes dip-coated with the thiol-ene material, hereby setting up a chemistry-controlled comparison. Neuronal survival around the larger thiol-ene probes appears to be consistently lower compared to the smaller dip-coated probes, at both 2 and 16 weeks after implantation. Thus, the differences in size of the two probes in the current study are the most likely driver for the heightened inflammatory response of the thiol-ene probes.

Our study adds to the current body of research which suggests that size of the implant is a very important consideration in intracortical microelectrode design and too large of an implant can counteract benefit conferred by improved material selection or approaches to target the biology (Seymour and Kipke, 2007). Based on these and other findings from the field, size of the implant needs to be a major consideration for electrode design.

The current study further highlights the importance of CD14 for microglial and macrophage responses to DAMPs. As previously reported, CD14 is central for microglial responses to damage signals in the brain (Janova et al., 2016). In this current study, *Cd14^-/-^* resulted in higher neuronal density (Figures 2A,C) and decreased glial scar (Figures 3A,C) at 2 weeks post implantation, further revealing the importance of CD14 as a molecular target to reduce neuroinflammation. In our past work, we have demonstrated that partial inhibition of CD14 (in myeloid cells) was shown to improve single-shank, multi-channel electrode performance over time, whereas a complete CD14 inhibition did not result in such promising electrode performance (Bedell et al., 2018). In the current study, coupling complete inhibition of CD14 with the thiol-ene probe not only prevented exaggerated frustrated phagocytosis but also inhibited proper wound healing; the promising effects afforded by targeting CD14 were not able overcome the detriments of a larger probe size. However, the promising results of partial CD14 inhibition suggest that controlled of the thiol-ene inhibition of CD14 with a drug may still be promising for the integration of softer probes.

The dimensions of the thiol-ene probe are larger than that of the silicon probe, likely contributing to the increased inflammation seen on the thiol-ene probes compared to the uncoated silicon probes. The length of the thiol-ene probe taper (1.4 mm) is greater than that of the uncoated silicon probe taper (0.6 mm). However, the angle of their tips are similar, at 41.6° and 47°, respectively. As such, the differences in the angle between the tips are not expected to contribute significantly to vascular damage and hence tissue response; it is unlikely to affect our study. It also should be noted that there are conflicting reports in the literature to the benefits/disadvantages of different insertion speeds. Although Bjornsson et al. (2006) report that *fast* insertion (2 mm/s) results in less vascular damage and tissue strain, there are other reports indicating that slower insertion speeds afford time for tissue to adapt to probe thus decreasing compressive forces (Edell et al., 1992; Andrei et al., 2011).

Together, the results found in the current study suggest that large cross-sectional area probes comprised of a minimally swelling, yet softening material cannot overcome inflammation driven by large penetration profile differences. Accordingly, the differences in the size of the electrodes is a major limitation of our study. Overall, the results presented here highlight the detriment of only considering one or two aspects of probe design and mitigation of biological response. When approaching translatable strategies to improve chronic intracortical microelectrode performance by decreasing inflammation, one needs to consider many characteristics in tandem.

## Conclusion

The current manuscript demonstrates the impact of size of the probe on the initial stages of inflammation. To reduce inflammation, the cross-sectional area of the probe should be minimized. The current study also characterizes the acute benefits of targeting CD14 and further confirms the TLR/CD14 pathway as a mechanism amenable for therapeutic targeting. In the initial weeks after probe implantation, the benefits a minimally swelling soft probe affords does not exceed the inflammation driven by a probe with 4× the cross-sectional area. When optimizing probe design for intracortical microelectrodes, many elements of the probe need to be carefully considered, especially size.

## Materials and Methods

### Electrodes

**FIGURE 6:**
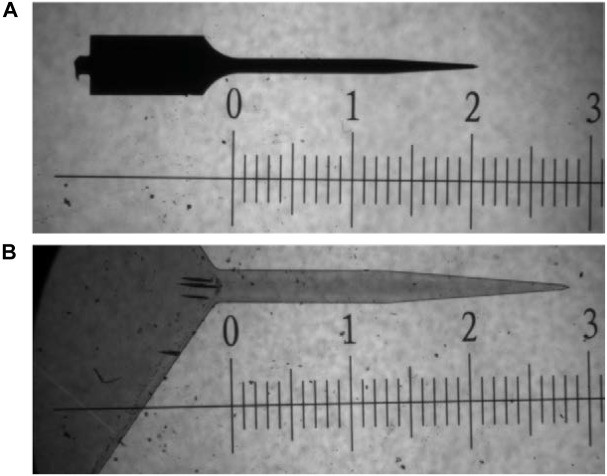
Bright field microscope image comparing dimensions of probes used in study. **(A)** uncoated silicon probe and **(B)** thiol-ene probe. Both images are taken at 5× magnification. Scale is in mm.

Single shank, uncoated, Michigan style silicon probes (2 mm × 15 μm × 123 μm; 47° taper angle) and thiol-ene based shape memory polymer (SMP) probes (3 mm × 30 μm × 290 μm; 41.6° taper angle) were used as intracortical probes (Figure 6). Polymer films were fabricated as previously described (Ecker et al., 2017). Briefly, 0.5 mol% 1,3,5-triallyl-1,3,5-triazine-2,4,6(1H,3H,5H)-trione (TATATO), 0.45 mol% trimethylolpropane tris(3-mercaptopropionate) (TMTMP), and 0.05 mol% Tris[2-mercaptopropionyloxy)ethyl] isocyanurate (TMICN) were mixed with 0.1 wt% of photoinitiator 2,2-dimethoxy-2-phenylacetophenone (DMPA). The polymer solution was then spin coated on glass slides using a spin coater (Laurell WS-650-23B) to receive ∼33 μm films before they were cured for 2 h at 254 nm (UVP CL-1000 cross-linking chamber) followed by an overnight post-cure at 120 °C under vacuum. Dummy probes were fabricated in the UT Dallas Class 10000 cleanroom facility. The 33-μm SMP-on-glass substrates were used as the starting substrates in the cleanroom. Low temperature silicon nitride (using PlasmaTherm-790 PECVD) was deposited to act as a hard mask for the following plasma etching processes. The device outline/shape was then patterned using standard lithography techniques. The hard mask and the SMP layer were plasma etched in Technics RIE using SF_6_ and O_2_ plasma, respectively. After the 30-μm SMP layer was plasma etched down to the glass slide, the remaining silicon nitride hard mask was etched away in diluted 10:1 HF dip. A ∼3 μm SMP layer from the surface of these devices was etched in Technics RIE using O_2_ plasma. The devices were then released by soaking in DIW. The material is characterized by a glass transition temperature (*T*_g_) of 52°C before, and 35 °C after softening under physiological conditions as measured by dynamic mechanical analysis (TA RSA-G2). The storage modulus *E*′ at 37°C (measured in tension) changes from 1.2 GPa (dry) to 35 MPa (soaked). Stainless-steel wires (∼3 mm length) were used as dummy ground and reference wires to mimic the implants involved with functional probes. Probes and wires were sterilized *via* a cold ethylene oxide gas cycle as previously described.

### Animals

C57/BL6 (strain #000664) and *Cd14^-/-^* (C57/BL6 background) (strain #003726) were bred in-house. Strain of *Cd14^-/-^* mice was verified *via* genotyping according to the protocols established by the vendor (Jackson Laboratories). Both male and female mice between 8 and 12 weeks of age were used for surgeries. Prior to surgery, mice were group housed with food and water *ad libitium* while maintained on a 12-h light/dark cycle. After surgery, mice were individually housed. All animal handling was performed in a class II sterile hood using microisolator techniques. All procedures and animal care practices comply with the protocol approved by the Case Western Reserve University Institutional Animal Care and Use Committee.

### Surgical Implantation of Electrodes

A total of three holes were drilled in the exposed skull using a 0.45 mm size bit (Stoetling Co.) with adequate breaks in the drilling pulses to prevent overheating of the skull (Shoffstall et al., 2018b). The probe hole was created in the skull over the motor region of the brain (1.5 mm lateral and 0.5 mm anterior to bregma) (Tennant et al., 2011). Two additional craniotomies were conducted for dummy ground and reference wires which were implanted in the contralateral hemisphere to the probe (∼2 mm lateral, ∼2 mm rostral and caudal to bregma). The dummy probes and dummy wires were manually inserted (∼2–3 mm/s) into the cortex. Silicone elastomer (Kwik-Sil, World Precision Instruments) and dental acrylic (Fusio/Flow-it ALC, Patterson Dental) tethered the probe and wires to the skull. The incision site was then sutured closed using 5-0 monofilment polypropylene suture. To minimize variability, the same surgeon performed all implantation surgeries.

### Immunohistochemistry

At each 2 and 16 weeks post-implantation, mice were sacrificed and brain tissue was harvested. At the respective time point, mice were anesthetized with an intraperitoneal injection of ketamine/xylazine cocktail. Each animal was then transcardially perfused with phosphate buffered saline (PBS) followed by 4% paraformaldehyde (PFA) to fix the tissue. Mouse heads were post-fixed for an additional 2 days in 4% PFA at 4°C. After fixation, brains were extracted and immersed in 30% sucrose for at least 48 h. After dummy electrodes and wires were removed, brain tissue was cryopreserved in optimal cutting temperature compound (OCT) (Tissue-Tek). Horizontal brain tissue sections (16 μm thick) were obtained using a cryostat and stored at -80°C.

To compare neuroinflammation and neuronal density in the area adjacent the implanted dummy shank among conditions, immunohistochemistry was utilized using previously established methodology (Potter et al., 2014). Only tissue slices between ∼320 and 1000 μm from the surface of the cortex were used as this depth corresponds with Layers III–VI of the mouse motor cortex, the layers from which functional probes aim to record (Oswald et al., 2013). After blocking the tissue (4% chicken serum, 0.3% Trition-X-100 in 1× PBS), the following primary antibodies (in 4% chicken serum, 0.3% Trition-X-100 in 1× PBS) were added to incubate overnight at 4 °C: Rabbit anti-GFAP (1:500, Z0334, Dako), mouse anti-neuronal nuclei (NeuN) (1:250, MAB377, Millipore), rat anti-CD68 (1:500, ab53444, Abcam), and rabbit anti-immunoglobulin G (IgG) (1:500, STAR26B, Bio-Rad). Visualization of the inflammatory and neuronal markers was achieved with respective Alexa Fluor^®^ secondary antibodies (1:1000) (in 4% chicken serum, 0.3% Trition-X-100 in 1× PBS). DAPI (Molecular Probes D3571) was incorporated in secondary antibody solution to visualize cell nuclei. Furthermore, tissue autofluorescence was reduced by incubating tissue sections with of 0.5 mM copper sulfate buffer solution for 10 min (Potter et al., 2012b). Finally, copper sulfate buffer was washed off thoroughly with MilliQ H_2_O, and slides were coverslipped using Fluoromount-G. Slides were stored in the dark at 4 °C until imaged.

### Imaging and Quantitative Analysis

Images were acquired using a 10× objective on a Carl Zeiss AxioObserver.Z1 (Zeiss Inc.) inverted epifluorescence microscope. Fluorescent markers were imaged on single optical sections using an AxioCam MRm monochrome camera with fixed exposure times for each marker.

Images of fluorescent markers were analyzed using SECOND, a custom-written MATLAB program previously used in the Capadona lab (Goss-Varley et al., 2017). The fluorescent intensity of each marker in concentric rings at fixed distances (normalized by area) from the probe–tissue interface was measured as a function of distance from the implant. Prior to measurement, the user defines the implant hole and any imperfections in the brain slice to omit from the analysis. For each slice, raw fluorescent intensities were then normalized to background signal, defined as the fluorescent intensity of the concentric ring 600–650 μm from the interface. The area under the curve (AUC) was calculated from the intensity profile.

Neuronal densities at the interface were determined using AfterNeuN, another custom-written MATLAB program. Using AfterNeuN, the user manually defines the electrode implant region, any areas to be excluded from analysis, and neuronal cell bodies. The program then outputs the density of neurons at fixed radial distances from the electrode interface. Neuronal densities at uniform binned distances (50 μm bins) were then normalized to background counts from the same brain tissue slice 300–350 μm away from the interface.

### Immunohistochemistry Statistical Analysis

Table 1 indicates number of animals for each condition at each time point. Measurements from all brain tissue slices for a given animal were first averaged together (four to six brain slices per animal, average of 5.29 ± 0.87). Average intensity or count for a given condition was calculated using independent animal averages. Statistical analyses for the first experiment, comparing thiol-ene + *Cd14^-/-^* to silicon shank + WT were performed using unpaired *t*-tests. All statistical analyses assessing immunohistochemical results comparing all four conditions were performed using a general linear model with a two-way analysis of variance (ANOVA) using Minitab software with genotype (WT or *Cd14^-/-^*) and electrode material (uncoated silicon or thiol-ene) as separate factors. Results were considered significant at *p* < 0.05 and expressed as mean ± standard error of mean.

**Table 1:** Number of experimental animals for each condition at each time point.

	2 weeks post implantation	16 weeks post implantation
Silicon shank + WT	10	6
Thiol-ene + WT	10	4
Silicon shank + *Cd14^-/-^*	8	6
Thiol-ene + *Cd14^-/-^*	9	4

## Author Contributions

HB, WV, JP, and JC contributed substantially to the conception or design of the work, analysis, and interpretation of data for the work, drafting, and revising the manuscript for important intellectual content, approved the final version to be published, and agreed to be accountable for all aspects of the work. HB, SS, XL, EM, AJS, and SL aided in the collection and analysis of histological data. AS, ME, and VD fabricated and characterized the SMP probes. All authors approved the final version to be published and agreed to be accountable for all aspects of the work.

## Conflict of Interest Statement

The authors declare that the research was conducted in the absence of any commercial or financial relationships that could be construed as a potential conflict of interest.
