# Supplementary material for: Understanding the Effects of Both CD14-Mediated Innate Immunity and Device/Tissue Mechanical Mismatch in the Neuroinflammatory Response to Intracortical Microelectrodes
Source: Front Neurosci. 2018 Oct 31;12:772. doi: 10.3389/fnins.2018.00772 (PMC6220032; doi:10.3389/fnins.2018.00772)
Supplement: Supplementary file 1 [file Data_Sheet_1.docx]

**Supporting Information (SI)**

**Understanding the effects of both CD14-mediated innate immunity and device/tissue mechanical mismatch in the neuroinflammatory response to intracortical microelectrodes**

Hillary W. Bedell^1,2^, Sydney Song^1,2^, Xujia Li^1^, Emily Molinich^1^, Shushen Lin^1^, *Allison Stiller^3^,* *Vindhya Danda ^3,6^, Melanie Ecker ^3,4,6^, Andrew J. Shoffstall*^1,2^, Walter E. Voit^3,4,5,6^, Joseph J. Pancrazio^3^, Jeffrey R. Capadona^1,2*^

*^1^Department of Biomedical Engineering, Case Western Reserve University, School of Engineering, 2071 MLK Jr. Drive, Wickenden Bldg, Cleveland OH 44106, USA*

*^2^Advanced Platform Technology Center, L. Stokes Cleveland VA Medical Center, Rehab. R&D, 10701 East Blvd. Mail Stop 151 AW/APT, Cleveland OH 44106, USA*

*^3^Department of Bioengineering, The University of Texas at Dallas, Richardson, TX, USA;*

*^4^Department of Materials Science and Engineering, The University of Texas at Dallas, Richardson, TX, USA;*

*^5^Department of Mechanical Engineering, The University of Texas at Dallas, Richardson, TX, USA;
^6^Center for Engineering Innovation, The University of Texas at Dallas, Richardson, TX, USA;*

**Denotes corresponding author*

*Jeffrey R. Capadona, Ph.D.*

*Case Western Reserve University*

*2071 Martin Luther King Jr. Drive b*

*Cleveland, OH, 44107*

Email: [jrc35@case.edu](mailto:jrc35@case.edu)

**Supplementary Results:**

As the following data was generated from separate experimental groups consisting of rats (versus mice used in the primary manuscript), the methods and results are presented here as Supplementary Data. Male Sprague Dawley rats (200-250g, n=11 per group) were implanted with either stiff silicon microelectrode probes dip-coated with shape memory polymer or thiol-ene based SMP probes. Due to manufacturing constraints, the dimensions of the thiol-ene probes were ~30x290 µm in cross-section compared to the dip-coated probes which were ~30x130 µm. Therefore, our experiment comprised a chemistry-matched comparison of the two groups. As performed previously by our group and others, microelectrode probes were implanted bilaterally (one in each hemisphere) and treated as independent of one another [[1](#_ENREF_1), [2](#_ENREF_2)]. After implantation, animals were housed for 2 or 16 weeks, representing initial and late onset neurodegeneration [[3](#_ENREF_3), [4](#_ENREF_4)]. Neuronal density (NeuN) was assessed by the same methods presented in the manuscript. Neuronal survival around the larger thiol-ene probes appears to be consistently lower compared to the smaller dip-coated probes, at both 2 and 16 weeks after implantation (**Figure S1**).

*
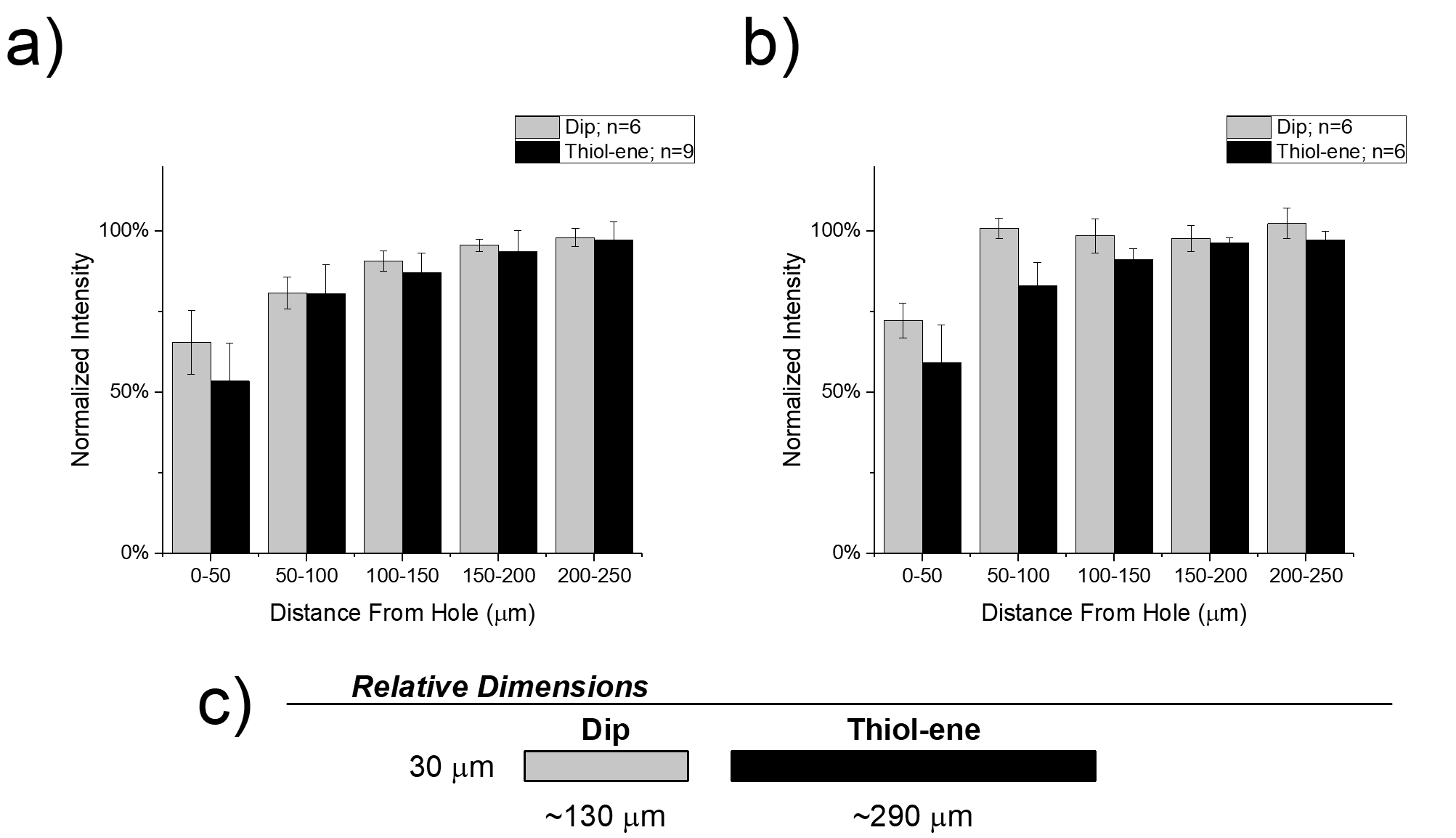
*

**Figure S1. Immunohistochemical evaluation of neuronal density.** Neuronal density evaluated as NeuN+ cells with respect to distance from the explanted microelectrode hole (μm). A) 2 week. B) 16 week post implantation. C) Relative dimensions of two different probes used in study.

**Supplementary Methods:**

*Dip-coating*

The thickness of the thiol-ene layer was verified to ensure evenly coated surfaces using a scanning electron microscope (SEM) (Zeiss Supra 40 and Zeiss EVO LS 15 Scanning Electron Microscopes, Zeiss, Inc.) on several representative devices. The coating profile was consistent across various sampled probes; however, the thickness of the coating varies over the length of the probe, with the coating being slightly thicker at the base than the tip. The mean thickness along the length is ~8 µm with a range of 2–24 µm. The surface modification of the bare silicon shank requisite to add the thio-lene coating reduced the thickness of the probes down to about 14 µm; thus, the final thickness of the coated probes were 30 µm. Although the coating along the probe was not consistent, overall probe volume was still similar to that of uncoated silicon probes.

*Device Implantation*

All procedures were reviewed and approved by the Louis Stokes Cleveland Department of Veterans Affairs Institutional Animal Care and Use Committee. Sprague Dawley rats were anesthetized (3-5%) and kept under anesthesia (1-3%) using an isoflurane vaporizer to maintain a surgical plane of anesthesia. Once anesthetized, eye lubricant was applied and the fur on the scalp was shaved and cleaned. Prior to surgery, the rats received 16 mg/kg cefazolin and 1 mg/kg meloxicam subcutaneously as prophylactic antibiotic and analgesic, respectively. Additionally, a single dose of 0.2 ml of 0.25% bupivacaine (local anesthetic) was administered subcutaneously at the incision site. The surgical site was cleaned in triplicate with betadine followed by isopropyl alcohol scrubs. Surgery was performed under an operating microscope. Craniotomies were performed carefully with a combination of intermittent pausing and saline application, to prevent overheating from drilling [[5](#_ENREF_5)]. A sterile ruler and forceps were used to mark the area to be drilled, 2 mm lateral to midline, 3 mm posterior to bregma (corresponding to a region of the sensory cortex). Removal of the final thinned bone flap was performed with ultrafine rongeurs to prevent incidental mechanical damage to the brain from the drill tip. After careful reflection of the dura, microelectrodes were implanted ~2 mm deep by hand using micro-forceps, avoiding superficially visible vasculature. Kwik-Cast was applied to cover the craniotomy and allowed to cure, followed by application of cold-cure dental acrylic to build up a stable cement base around the implant. Given the low profile of the dummy probe implants (e.g., as compared to functional recording microelectrodes that require an exposed head-stage), skin was sutured together and treated with non-prescription triple-antibiotic cream. Post-operative analgesic was provided for 2 days following implantation (1 mg/kg meloxicam q.d.) and post-operative prophylactic antibiotics were provided for 1 day following implantation (16 mg/kg cefazolin, b.i.d.). There were no complications with post-operative infection or observations of overtly unmanaged pain from the procedure.

Tissue Extraction and Preparation

At the pre-determined end points (2 or 16 weeks), animals were perfused transcardially under deep anesthesia to prepare the tissue for histological processing. After achieving deep plane of anesthesia, using a ketamine/xylazine cocktail (80 mg/kg and 10 mg/kg respectively), rat aortas were cannulated with a gavage needle via an incision in the left ventricle and connected to a perfusion pump. Phosphate buffered saline (1x) was perfused until the fluid exiting the excised vena cava/right atrium appeared clear. Tissue was then fixed by perfusion with ~200 ml of 4% w/v paraformaldehyde solution. The tissue was post-fixed in 4% w/v paraformaldehyde solution overnight. After careful extraction, brains were subsequently cryoprotected with a gradient of sucrose (with 0.1% sodium azide) from 10-30% w/v, and frozen in OCT blocks and stored at -80**°** C until sectioning. Tissue sections, 20 µm thick, were generated on a cryostat and were collected on Fisherbrand ‘Superfrost Plus’ glass slides. Staining, imaging and subsequent analysis was otherwise performed exactly as presented in the manuscript.

**References**

[1] H.C. Lee, J. Gaire, S.W. Currlin, M.D. McDermott, K. Park, K.J. Otto, Foreign Body Response to Intracortical Microelectrodes Is Not Altered with Dip-Coating of Polyethylene Glycol (PEG), Frontiers in neuroscience 11 (2017) 513.

[2] G. Lind, L. Gällentoft, N. Danielsen, J. Schouenborg, L.M. Pettersson, Multiple implants do not aggravate the tissue reaction in rat brain, PloS one 7(10) (2012) e47509.

[3] K.A. Potter, A.C. Buck, W.K. Self, J.R. Capadona, Stab injury and device implantation within the brain results in inversely multiphasic neuroinflammatory and neurodegenerative responses, J Neural Eng 9(4) (2012) 046020.

[4] G.C. McConnell, Chronic inflammation surrounding intra-cortical electrodes is correlated with a local neurodegenerative state, Biomedical Engineering, Georgia Institute of Technology, Atlanta, GA, 2008, p. 153.

[5] A.J. Shoffstall, J.E. Paiz, D.M. Miller, G.M. Rial, M.T. Willis, D.M. Menendez, S.R. Hostler, J.R. Capadona, Potential for thermal damage to the blood-brain barrier during craniotomy: implications for intracortical recording microelectrodes, J Neural Eng 15(3) (2018) 034001.
